# Healthier Supermarkets: Aligning Retailer and Consumer Perspectives on Evidence-Based Strategies to Improve Purchase Behaviour

**DOI:** 10.3390/foods15193412

**Published:** 2026-09-24

**Authors:** Paige G. Brooker, Emily Brindal, Amanda Watson-Tran, Gilly A. Hendrie

**Affiliations:** 1 Health and Biosecurity, Commonwealth Scientific and Industrial Research Organisation (CSIRO), Adelaide, SA 5000, Australia; 2 Preventive Health SA, Adelaide, SA 5000, Australia

**Keywords:** supermarket, retail food environment, public health, health promotion, consumer behaviour, implementation

## Abstract

Background/Objectives: Despite promising evidence for the influence of in-store supermarket initiatives to improve the healthiness of retail food environments, both retailer and consumer perspectives have not been examined simultaneously. The objectives of this research were to examine evidence-based initiatives through (1) measuring retailer perspectives on factors likely to influence uptake; (2) collecting consumer perspectives on which are most likely to promote healthier purchasing behaviour; and (3) synthesising findings to identify ‘win-win-win’ strategies across evidence, and stakeholder perspectives. Methods: Initiatives were drawn from scientific evidence across five overarching strategies: pricing, proximity, prompting, promotion, and healthy default picks. Retailers additionally assessed a sixth category, ‘promotion plus’, comprising multi-component initiatives that combine promotion with another strategy; this category was not assessed by consumers. Data for Part 1 used an online survey to capture perspectives of supermarket retail decision-makers (n = 5; treated as exploratory) on effectiveness, feasibility, scalability and sustainability of initiatives. Part 2 used a computer-assisted telephone survey of South Australian adults sampled through a population health survey (‘consumers’; n = 2877) to capture consumer perspectives on a subset of eight of these initiatives, adapted for the consumer shopping context. Consumers rated the perceived likelihood that each initiative would encourage healthier purchasing. In Part 3, data were triangulated into a matrix representing best cases across evidence and stakeholder groups. Results: Pricing and proximity were rated most favourably (69% to 87%, and 71% to 75%, respectively) across retailer and consumer perspectives and represent possible targets for voluntary retailer adoption. Prompting, promotion, and healthy default picks were rated more favourably by consumers (68% to 71%) than by retailers (50% to 60%). The retailer-only promotion plus category had the most promising evidence base but the lowest retailer rating (49%). Conclusions: Pricing and proximity strategies appear to offer the strongest ‘win-win-win’ opportunities to improve the healthiness of consumer purchasing behaviour, though retailer findings should be interpreted as exploratory given the small retailer sample. The alignment between evidence/retailer/consumer perspectives can guide researchers, policymakers, and retailers in prioritising initiatives with real-world potential.

## 1. Introduction

Dietary intake is influenced by a range of individual-, social-, environmental- and system-level factors [1,2,3]. Supermarkets are one actor in the system, with two-thirds of all food in Australia purchased from supermarkets [4] and similar figures in the US [5] and UK [6]. Supermarkets also actively influence purchasing decisions and, ultimately, population diets, through techniques typically referred to as the ‘four Ps of marketing’—product, price, placement, and promotion [7,8]. The literature provides examples of implementation of such strategies—such as reducing/replacing unhealthy foods for sale with healthier foods (product); using price reductions to increase acceptability of healthier foods (price); placing healthy checkout aisles in stores to shift the healthy/unhealthy balance (placement); and highlighting healthy options by displays, labels and samples to taste (promotion) [8]. These techniques can shape purchasing behaviour, particularly unintended, unhealthy purchases, even among consumers who make written or mental shopping lists [9]. There is growing evidence to suggest that changing the retail food environment to be more health-enabling via in-store interventions is possible.

A systematic overview of reviews and evaluation of primary articles examining the effectiveness of real-world supermarket-based interventions found that promotional strategies paired with another strategy appeared most promising for increasing sales of healthy foods among shoppers. Pricing strategies also appeared promising, particularly among low-income or rural population groups [10]. However, these initiatives were often researcher-led or initiated, and few have been delivered in real-world settings at scale [11]. To achieve the reach and scale required for positive population impact, retailers need to be consulted as a key stakeholder of implementation.

Retailers’ decision to implement healthy eating initiatives in-store may be influenced by business outcomes such as commercial viability, consumer preferences and trends, and community outcomes [12]. Retailer decision-making is further complicated by upstream commercial relationships with manufacturers, in which suppliers, particularly large suppliers, actively shape shelf space through ‘category management’ arrangements. These arrangements can result in a disproportionate dominance of the largest, processed food brands [13], suggesting that even if retailers were willing to implement healthy eating initiatives, structural commercial pressures may limit their ability to do so. A discrete choice experiment with Australian supermarket retailers found that customer satisfaction was the most valued business outcome (ranked above net profit, supplier satisfaction, and ease of implementation), with retailers willing to pay up to AUD 32,136 per year for strategies that led to higher customer satisfaction [14]. Understanding the factors that influence implementation, scalability and sustainability of retail initiatives, and how these factors might interact to influence retailers’ decision-making, is critical [15]. Co-designed approaches in which researchers and retailers develop initiatives together have shown promise. For example, the EatWell@IGA trial demonstrated that multi-component in-store interventions are feasible when built around existing retailer practices and relationships [16].

Consumers are another key stakeholder group that could influence the success of these initiatives. Alsubhi and colleagues conducted a systematic review of studies to explore consumer willingness to pay for healthier food products. Nearly 90% of the experiments included in their review (n = 23/26) found consumers would pay a price premium for healthier food products (mean +31%; range 5% to 92%), with consistency among studies reporting a positive willingness to pay for reduced-fat products and foods higher in fruit and vegetables [17]. This suggests that there is consumer demand for healthier options and this may be a case for retailer adoption of health-enabling strategies. Public support for healthy supermarket initiatives is also generally high. A cross-sectional analysis of over 22,000 adults across five countries found that the majority supported placement-based initiatives, such as healthy-only checkouts and expanded shelf space for healthier foods, though the level of support varied by initiative type and by country [18]. Given more than two-thirds (68%) of food and beverage-related expenditure [19] is in supermarkets and grocery stores, understanding consumer perspectives on which evidence-based initiatives would most influence their purchasing behaviour is essential for effective intervention design.

The current evidence base has examined retailer perspectives and consumer preferences separately, and not with direct comparison of the same strategies. For example, Blake and colleagues identified commercial viability and stakeholder perceptions as critical implementation factors in their scoping review of healthy food retail evaluations, but the heterogeneity of included interventions prevented direct comparison of how retailers and consumers prioritise specific strategies [12]. A process evaluation of the Eat Well@IGA trial captured retailer, staff and customer perspectives on a co-designed multi-component intervention [20], but was not designed to compare discrete strategies prospectively. No published study has simultaneously and systematically assessed retailer and consumer perspectives on the same set of evidence-based strategies within a single programme of research—a gap that prevents identification of stakeholder alignment, or misalignment, for specific, comparable interventions.

Considering scientific evidence, retailer perspectives and consumer perspectives together is important because a strategy is only likely to be both implemented and effective if all three conditions hold, that is, the evidence supports it, retailers regard it as feasible and worthwhile, and consumers are receptive to it. Bringing them together for the same underlying set of evidence-based initiatives allows strategies to be prioritised according to their real-world potential rather than their evidence base alone. Therefore, the objectives of this study were to: (1) investigate retailer perspectives on the effectiveness, feasibility, scalability, and sustainability of evidence-based healthy supermarket initiatives; (2) investigate which evidence-based initiatives consumers perceived to be most influential in encouraging healthier food and beverage purchases; and (3) synthesise findings to identify ‘win-win-win’ opportunities where there is alignment between evidence-based strategies, and support from both retailers and consumers.

## 2. Methods

### 2.1. Overview

This research was conducted in South Australia between November 2023 and April 2024. Part 1 was an online survey of supermarket retailers; Part 2 was a telephone survey of South Australian adults (‘consumers’). Both surveys evaluated evidence-based healthy supermarket initiatives which were identified from a systematic overview of reviews examining real-world supermarket interventions [10]. The initiatives spanned five strategy types based on the framework developed by Kraak et al. [21] and adapted for grocery store settings by Slapø et al. [22]: pricing, proximity, prompting, promotion, and healthy default picks. A sixth category, ‘promotion plus’, was used for multi-component initiatives combining promotion with another strategy. The five strategies form the basis for comparison between retailers and consumers throughout; promotion plus was assessed by retailers only. All initiatives had been tested in physical or online supermarket settings for their effectiveness in improving the healthiness of consumer purchases.

### 2.2. Ethical Approval and Consent to Participate

Data collection and initial analysis for Part 1 were approved by the CSIRO Health and Medical Human Research Ethics Committee (2023_051_LR). Data collection and initial analysis for Part 2 were approved by the Department for Health and Wellbeing Human Research Ethics Committee (HREC/18/SAH/78—2020/HRE00418), with reciprocal approval from the CSIRO Health and Medical Human Research Ethics Committee (2024_026_RR). Re-analysis of these data for the purposes of this publication was approved by the CSIRO Health and Medical Human Research Ethics Committee under a secondary analysis application (H-2026-0353). All research was conducted in accordance with the Declaration of Helsinki. Prior to undertaking the surveys, all participants provided informed consent to their data being used for research purposes via a digital check box.

### 2.3. Part 1: Retailer Perspectives

#### 2.3.1. Participants and Recruitment

Individuals were eligible to participate if they were: (i) ≥18 years of age; (ii) currently employed by, or owned, a supermarket in Australia; and (iii) in a decision-making role influencing product marketing, pricing, and placement of food products. These may include Level I (corporate level) personnel, who are responsible for strategic planning and establishing criteria for store image, customer service, store layout, store locations or management organisations, or Level II functions, which focus on the purchase and distribution of goods to retail stores, and coordination of merchandising and promotional activities [23].

An invitation email containing the participant information sheet and consent form was sent to potential participants identified by the research team. Following the initial invitation, up to two follow-up reminder emails were sent. Other key personnel were identified through snowball sampling; each ‘key contact’ was asked to invite two to three of their colleagues who also met eligibility criteria and were willing to participate. Participation was voluntary and no incentives were offered.

#### 2.3.2. Measures

The online survey was hosted on the Qualtrics XM Platform™ (Provo, UT, USA) and was open from November 2023 to April 2024. Retailers were presented with 19 evidence-based initiatives, informed by previous research [10], across five broad strategies, namely, pricing (n = 3), proximity (n = 3), prompting (n = 3), promotion (n = 3), and healthy default picks (n = 2), along with the additional promotion plus category (n = 5). Multi-component ‘promotion plus’ initiatives were defined as those combining a promotional initiative with another strategy (e.g., a price discount combined with consumer education). Healthy default picks included two initiatives tested in online supermarket settings. For each initiative, respondents were first asked whether their workplace had previously tried the initiative (yes/no/unsure), they then rated each initiative using a six-point Likert scale from 1 = ‘not very…’ to 6 = ‘very…’: for its (i) ease of implementation; (ii) effectiveness in improving the healthiness of consumer purchases; (iii) scalability across multiple stores; (iv) longer-term viability; and (v) willingness to try the initiative (or try again if previously implemented).

Respondents were also asked to rank the relative importance of four factors (effectiveness, scalability, longer-term viability, ease of implementation) when considering whether to implement a new initiative in-store. Finally, respondents who wanted to improve the healthiness of their purchases were also asked to select the single initiative they would most like to see implemented if they could choose only one.

#### 2.3.3. Data Analysis

Data were analysed using IBM SPSS (Version 29.0.1, Armonk, NY, USA). Descriptive statistics (frequencies, mean, standard deviation, range) were used to analyse survey responses. Initiatives were grouped according to their overarching strategy type (pricing, proximity, prompting, promotion, healthy default picks), with promotion plus analysed as a separate sixth category. The results are reported at both strategy and initiative level. Incomplete responses were excluded from the final analysis.

### 2.4. Part 2: Consumer Perspectives

#### 2.4.1. Participants and Recruitment

Consumer insights were obtained through a computer-assisted telephone interview (CATI) survey of South Australian adults, conducted as part of the Population Health Survey Module System (PHSMS). The PHSMS survey is an omnibus-type service managed by Preventive Health SA’s Epidemiology Branch to support the collection of data concerning the health and wellbeing of the South Australian community.

Individuals were eligible if they were: (i) aged 18 years or over; (ii) resided in South Australia; and (iii) had access to a telephone (mobile device or landline). The survey was designed to take approximately 20 min to complete. Using random digit dialling, a dual-frame overlapping sampling method of mobile and landline devices was used to recruit and collect data from a representative sample of South Australian adults across metropolitan and rural areas.

#### 2.4.2. Measures

The survey included questions on demographic characteristics (age, sex, area of residence, marital status, employment status, highest level of education completed, and number of children in household), followed by questions about respondents’ experience with supermarkets, including their:

- Shopping behaviours: Respondents were asked about how (online/in-store), how often (frequency of visits; daily to never), who they shop with, and which supermarket(s) they shop at. Respondents were also asked to rate how much they thought the supermarket environment (store layout, products available, promotions) influenced what foods and beverages they buy, using a five-point scale (1 = ‘to a great extent’ to 5 = ‘not at all’).
- Desire to change purchasing behaviour: Respondents were asked whether they wanted to improve the healthiness of foods and beverages they buy (yes/no/don’t know), and (if ‘yes’) which food categories they would most like to change their purchasing behaviours of.
- Perceptions of initiatives: From the 19 evidence-based initiatives tested with retailers, 8 relevant to the consumer shopping context were presented, spanning pricing (n = 1), proximity (n = 3), promotion (n = 2), prompting (n = 1), and healthy default picks (n = 1). No promotion plus initiatives were presented to consumers, as these combine multiple strategies and are considered not to be encountered by shoppers as a discrete, identifiable intervention. Respondents who wanted to improve the healthiness of their purchases were asked to rate how likely each initiative would be to encourage them to purchase healthier food and beverages; those who did not were asked how likely it would be to encourage others. Both groups used a five-point scale (1 = ‘very likely’ to 5 = ‘very unlikely’). Respondents who wanted to improve the healthiness of their purchases were also asked to select the single initiative they would most like to see implemented if they could choose only one. Respondents who were unsure whether they wanted to change their purchasing were not asked to rate the initiatives. A full comparison of the presentation of initiatives to retailers versus consumers is presented in Supplementary Table S1.

A copy of the interview questions related to supermarket purchasing behaviour is available in the Supplementary Materials.

#### 2.4.3. Data Analysis

Data were weighted to the South Australian population using a raking technique incorporating population characteristics (gender, age, area of residence, country of birth, dwelling status, marital status, education level, employment status, household size), with benchmarks derived from the June 2021 ABS Census data. Prior to analysis, initiative rating scores were reverse-coded so that higher scores reflected greater perceived likelihood to influence purchasing behaviour (i.e., 1 = very unlikely to 5 = very likely). ‘Don’t know’ and ‘prefer not to say’ responses were excluded separately for each initiative, so the number of respondents contributing to each rating varied. Mean scores were expressed as a percentage of the maximum possible score (mean/5 × 100). Data were analysed using IBM SPSS (Version 29.0.1, Armonk, NY, USA). Descriptive statistics (weighted counts, percentages, mean, standard deviation) were used to summarise study findings.

### 2.5. Part 3: Evidence/Retailer/Consumer Triangulation

Alignment between consumer and retailer perspectives on the evidence-based strategies was presented in a matrix to show a summary of stakeholder perspectives for each strategy. Because several initiatives were adapted or consolidated for the consumer survey (Supplementary Table S1), alignment was assessed at the level of the overarching strategy rather than the individual initiative. The strength of the scientific evidence for each strategy was derived from a systematic overview of reviews [10]. Only initiatives that had been effective in changing consumer purchasing behaviour were included.

Retailer perspectives of the strategies were derived from a composite of five factors (ease of implementation, effectiveness, scalability, longer-term viability and willingness to try), reflecting the multidimensional nature of real-world implementation decisions [12]. A mean score was created out of a possible 6 and expressed as a percentage (mean score/6 × 100). Consumer perspectives reflected the perceived influence strategies had on purchasing behaviour, expressed as a percentage of the maximum possible score (/5). Retailer and consumer scores were derived from different constructs and may not be directly equivalent; retailer perspectives were a composite of five implementation-relevant factors, whereas consumer perspectives reflect a single factor.

The strength of retailer and consumer perspectives was categorised into three bands: higher, more positive, perspectives (percentage score ≥ 67%); moderate (>33–<67%); lower (≤33%). These thresholds were applied a priori rather than derived from the observed data. Thresholds based on the observed scores would have been unstable given the small retailer sample, and using the full range allowed both sets of scores to be expressed on the same scale. Retailer and consumer perspectives of the strategies were then classified as aligned (if retailer and consumer perspectives fell within the same band, e.g., Aligned—Higher Support), or misaligned (if retailer and consumer perspectives fell in different bands, e.g., Consumer—Higher Support/Retailer—Moderate Support). Because the two scores are not directly equivalent, ‘alignment’ describes convergence in the relative positioning of each strategy within each stakeholder group rather than equivalence of scores. Promotion plus was not included in the matrix, as consumer data were not collected for this category; the matrix therefore presents the five strategies assessed by both stakeholder groups. The matrix is intended as a visual summary of stakeholder alignment rather than a statistical comparison and should be treated as exploratory.

## 3. Results

### 3.1. Part 1: Retailer Perspectives

#### 3.1.1. Participant Characteristics

In total, 27 retail staff were invited to participate. Seven individuals commenced the online survey (26% response rate); five completed the survey and were included in the final analysis (19% completion rate). The retail staff were a mix of general managers, marketing and operations managers.

#### 3.1.2. Prior Experience with the Initiatives

Retail staff (n = 5) reported that their supermarket had tried an average of 8 of the 19 initiatives (range: 4–14). Most initiatives had been tried by at least one respondent (79%, 15/19). Prior experience was most common for proximity and pricing initiatives and least common for healthy default picks, which no respondent had tried. Composite scores and prior experience for each of the 19 initiatives are reported in Supplementary Table S2.

#### 3.1.3. Retailer Ratings of Evidence-Based Initiatives

Retailers rated each initiative on ease of implementation, effectiveness, scalability, longer-term viability, and their willingness to try the initiative (or try again). Pricing initiatives scored highest for ease of implementation (86%), while proximity initiatives scored highest for effectiveness (84%), as well as willingness to try (77%), longer-term viability (76%), and scalability (71%; Supplementary Figure S1). When asked to rank the relative importance of factors when considering whether to implement a new initiative, effectiveness was ranked as most important, followed by scalability and longer-term viability, with ease of implementation ranked as least important.

Retailer perspectives, as a composite score across all five factors, for individual initiatives and the average across each strategy is shown in Figure 1. Retailers’ perspective on proximity strategies was highest (75%), followed by pricing (69%), promotion (60%), and prompting (55%). Of the five overarching strategies, healthy default picks received the lowest composite score (50%); the retailer-only promotion plus category scored lowest overall (49%). Scores for each individual initiative are provided in Supplementary Table S2.

When asked to select their single most preferred initiative, three of five respondents chose proximity initiatives: placing fresh fruit and vegetables in prime locations (n = 2) or allocating more shelf space to fruit and vegetables (n = 1). The others (n = 2) selected a promotion initiative—advertising feature fruit and vegetables in the weekly store catalogue.

### 3.2. Part 2: Consumer Perspectives

#### 3.2.1. Participant Characteristics

A total of 3954 eligible South Australians were contacted, of whom 3001 completed the interview. A total of 124 respondents (4%) reported they ‘never’ shopped at supermarkets for food and beverages and were excluded from further analysis, resulting in a final sample of 2877 (Figure 2).

The mean age of respondents was 53 (±17) years, and just over half were female (n = 1569 of 2877; 54%). Most (n = 2108; 73%) were living in the metropolitan area, and there was a relatively even distribution across socioeconomic quintiles (Supplementary Table S3). Nearly nine in ten respondents shopped in-store (n = 2461; 86%), usually alone (n = 1983; 69%), and visited the shops 1–2 times/week (n = 1766; 61%). Nearly all had a preferred retailer (n = 2854; 99%), primarily chosen for convenience (Supplementary Table S4).

#### 3.2.2. Consumer Interest in Healthier Supermarkets

Perceptions of supermarket influence on purchasing behaviour were mixed: 44% (n = 1278 of 2877) felt it had little to no influence, 41% (n = 1176) felt the environment had some influence, and 15% (n = 422) were neutral or unsure.

Just over half of the respondents (n = 1604 of 2877; 56%) expressed interest in improving the healthiness of their grocery purchases. Among these, vegetables (n = 1169; 73%) and fruits (n = 1050; 65%) were the most favoured additions to their shopping cart, while chocolate and confectionery (n = 800; 50%), sugary or artificially sweetened drinks (n = 687; 43%), and cakes and biscuits (n = 636; 40%) were items consumers most wanted to reduce (Supplementary Figure S2).

Respondents who did not want to change their purchasing (n = 1222 of 2877; 42%) predominantly felt they already ate well enough (n = 1075; 88%). Fifty (2%) participants did not know/preferred not to say whether they would like to change their purchasing behaviour and were excluded from further analysis.

#### 3.2.3. Consumer Ratings of Evidence-Based Initiatives

Respondents (n = 2826) were asked whether each initiative would influence their personal behaviour (if they expressed an interest in improving their own purchasing; n = 1604) or the behaviour of others (if they were not interested in improving their own purchasing; n = 1222). Ratings were consistent for influencing their own or others’ behaviours (within 6 percentage points for all initiatives, Supplementary Table S5), so combined responses are reported below.

Consumer perspectives of the strategies were high overall (≥67%), although perspectives on individual initiatives within each strategy varied (Figure 3). Pricing (a 10–20% discount on fruit and vegetables) was rated the most favourably of all initiatives presented (87%). Consumer perspectives on the other four strategies were similar, with mean scores ranging from 68 to 71%.

When asked to select the single initiative they would most like to see implemented, the pricing initiative was most preferred, with 40% (n = 635 of 1604) of respondents choosing ‘offering discounts or coupons on selected products’. This was followed by a proximity initiative—‘reducing the number of discretionary items at checkouts’, chosen by 15% (n = 248 of 1604) of respondents.

### 3.3. Part 3: Evidence/Retailer/Consumer Triangulation

Figure 4 brings together the descriptive data on retailer and consumer perspectives for the five overarching strategies assessed by both groups. Promotion plus is not included, as it was assessed by retailers only; a comparison of how the eight consumer initiatives map to the 19 retailer initiatives is provided in Supplementary Table S1. With the exception of proximity, consumer perspectives on each of the strategies were more favourable than those of retailers, although the small retailer sample limits confidence in the size or consistency of this pattern. Of the five strategies, retailer and consumer perspectives were aligned (Aligned—Higher Support) for proximity and pricing. There was misalignment on retailer and consumer perspectives on promotion, prompting and healthy default picks—with consumers’ perspectives in the higher band and retailer perspectives moderate. No strategy fell into the lower band of support for either group, reflecting the evidence-based pre-selection of initiatives, and therefore misalignment never stretched across two bands (i.e., high to low).

Some strategies sat close to the upper threshold. Retailer perspectives on pricing (69%) and consumer perspectives on promotion (68%) and prompting (69%) fell within a few percentage points of the 67% cut-point, and their classification would change under modest alterations to the threshold. The Aligned—Higher classification for proximity (retailer 75%, consumer 71%) and the moderate retailer classification for healthy default picks (50%) are less sensitive to the choice of cut-point.

## 4. Discussion

This study investigated retailer and consumer perspectives on evidence-based supermarket strategies to improve consumer purchases, synthesising findings in an evidence/retailer/consumer triangulation to identify ‘win-win-win’ opportunities that are supported by scientific evidence, viewed by retailers as feasible to implement, and acceptable to consumers. To our knowledge, this is the first study to assess and compare retailer and consumer perspectives on initiatives drawn from the same evidence base within a single programme of research. Perspectives were most aligned on initiatives within the proximity and pricing strategies with both consumer and retailers rating these highly. Promotion, prompting and healthy default picks had mixed alignment with consumers rating these highly and retailers moderately, though several of these classifications sit close to the band thresholds. On all but one strategy, consumer perspectives were perceived as more favourable than those of retailers. One possible explanation is that consumers assess strategies primarily in terms of perceived personal benefit, whereas retailers weigh up commercial risk, operational capacity, implementation cost, and contextual fit—factors that simultaneously influence willingness to adopt strategies, particularly for new or complex strategies [12,14,24]. Throughout, differences between retailer and consumer perspectives should be read as indicative patterns warranting further investigation with larger retailer samples, rather than as established differences between the two stakeholder groups.

Pricing was rated most favourably by consumers (87%), and retailers considered it the easiest strategy to implement (86%), making it a strong target for voluntary retailer adoption. These findings are consistent with published research. For example, a recent meta-analysis of supermarket-based trials found that a 20% price reduction resulted in a 17% increase in fruit and vegetable purchases [25]. The context of data collection is also relevant. Australia is experiencing its highest levels of inflation in over three decades [26], placing growing pressure on household grocery budgets. In this environment, discounts on fruit and vegetables may serve a dual purpose: improving diet quality while directly addressing affordability concerns. While the consumer and retailer case for pricing is strong, the commercial implications of sustained discounting warrant further consideration. It is also plausible that consumers may adapt to sustained (‘long-run’) price reductions over time, potentially diminishing their behavioural impact [27].

Retailers rated proximity initiatives highest for effectiveness, willingness to try, longer-term viability, and scalability; and it was the only strategy where retailer perspectives exceeded consumers (albeit only marginally). Retailer confidence in proximity may partly reflect the degree of operational control they have over in-store placement decisions. Qualitative research with supermarket retailers has found that while supplier planograms are influential at the planning stage, retailers report having the authority to modify placement arrangements as desired [28]. Proximity was also rated favourably by consumers, averaging 71% across the three initiatives, though there was notable within-strategy variation (64% to 76%). For consumers, proximity initiatives operate largely through automatic processes, and consumers may not consciously attribute behavioural change to placement cues [29]. Self-reported ratings of perceived influence may therefore not fully capture how these strategies affect purchasing behaviour. When asked which single initiative they would like to see implemented, 15% of consumers who wanted to improve their purchasing (n = 248 of 1604) chose ‘reducing the number of discretionary items at checkouts’, making it the second most preferred initiative, and aligned with their desire to reduce discretionary food purchases. This consumer endorsement for checkout reform is notable in the context of recent international policy developments [30]. Voluntary checkout food policies may be particularly viable for retailers because, unlike pricing, they do not directly compromise commercial margins and may even improve the customer shopping experience [31].

Prompting, promotion, and healthy default picks were rated more favourably by consumers than retailers. Prompting was rated highly by consumers (69%), whereas retailer ratings for ease of implementation (44%) and willingness to try (52%) were among the lowest of any strategy, albeit based on five respondents. Retailers in the current study also ranked ease of implementation as the least important factor in implementation decisions, consistent with the direction of findings from a larger discrete choice experiment with Australian supermarket retailers [14]. The practical barriers associated with shelf labelling (staff time, maintenance burden, and the cost of printed materials in a commercially competitive environment) remain real constraints on sustainability [15,20]. Digital shelf labels may offer a pathway to reducing these barriers as they are easier to update, scalable, and do not require ongoing physical maintenance [32].

Promotion showed notable variation within the strategy: catalogue advertising was rated highest by retailers (89%; and all had tried it) but lowest by consumers among promotion initiatives (64%). This apparent divergence may partly reflect who engages with supermarket catalogues. Research with Australian consumers found no significant relationship between health consciousness and catalogue usage, likely because most foods featured in catalogues are not healthy [33,34]. Conversely, education-based promotion was rated more favourably by consumers (71%) than by retailers (45%) in our study, which may indicate an underutilised opportunity for strategies that build consumer skills alongside environmental change.

For healthy default picks, consumers rated the strategy favourably (71%) but retailer perceptions placed it in the moderate support band (50%) and no retailers had previously tried this strategy. The two healthy default picks initiatives from the scientific evidence were framed in an online shopping context for retailers, whereas the consumer version of the initiative removed the online framing (given online grocery shopping in Australia is still relatively low). Online grocery retail is a rapidly growing but relatively new space for health-enabling intervention, with Australian online supermarket sales exceeding AUD 9.4 billion in 2023 [35]. Retailer uncertainty may reflect the relative novelty of the online grocery environment as a setting for health-enabling interventions compared with more established in-store strategies. Evidence suggests the online environment is receptive to healthy default interventions; an Australian randomised trial found that repositioning healthier products to the top of an online grocery store page significantly increased the nutritional quality of purchases without affecting affordability [36].

Promotion plus, the sixth category assessed by retailers only, was not included in the consumer survey. The retailer findings nonetheless warrant discussion given the apparent gap between evidence and practice, though the absence of consumer data means no three-way comparison is possible for this category. Despite having the strongest evidence base of any approach reviewed, with positive or promising outcomes in five out of six (83%) high-quality primary studies [10], promotion plus received the lowest retailer rating of any category, scoring below all five of the overarching strategies. This gap may reflect the greater operational complexity of multi-component strategies relative to single-component alternatives. Implementation research in food retail settings consistently identifies complexity as a structural barrier to uptake, with retailers tending to implement simpler, low entry point strategies before progressing to more resource-intensive approaches [16,24,28]. Notably, the promotion plus evidence largely comprised controlled trials which may not reflect the real-world constraints faced by retailers [24]. Co-creation approaches in which multi-component strategies are connected to existing or familiar retailer practices, and built incrementally, may offer a more viable pathway to implementation and help close the gap between evidence and practice [37]. This is consistent with broader findings from the food retail benchmarking literature, which suggest that food retail research rarely progresses beyond performance measurement to the implementation of recommendations and continuous improvement; the steps most likely to drive actual change in retailer practice [38].

International examples raise important questions about the relative merits of voluntary versus mandatory approaches, and about which specific interventions are likely to gain sufficient stakeholder support for voluntary adoption. The UK government implemented mandatory restrictions on volume promotions (i.e., 2-for-1 offers) and prominent placement of products high in fat, sugar, and salt, including checkout bans [30]. Similar regulatory approaches have been adopted in Chile and Mexico, where placement restrictions and front-of-pack labelling for unhealthy products form part of broader food environment legislation [39,40,41].

### 4.1. Strengths and Limitations

A major strength of this study was the large, representative sample of South Australian consumers (n = 2877) captured through a state-based, public health computer-assisted telephone survey, with respondents from both metropolitan and rural areas and relatively even distribution across gender and socioeconomic groups. The consistency of consumer ratings across self- and other-framing conditions (within 6 percentage points for all initiatives) suggests that responses were not substantially influenced by framing, strengthening confidence in the consumer findings. The novel aspect of this work was collecting and comparing consumer and retailer perspectives on these science-backed strategies. However, despite considerable recruitment effort, only five retailer representatives completed the survey, meaning that retailer findings should be considered exploratory. The retail staff were all managers, likely with responsibility and experience in implementing instore initiatives; however, the small sample prevented this research from any comparisons between retailer types, and findings should not be generalised to the broader retail sector. Given the small sample, retailer percentages should be interpreted as indicative estimates rather than precise values, and differences between retailer and consumer ratings (including where retailer perspectives appeared numerically lower) should be interpreted with caution rather than as confirmed disparities. Recruitment coincided with an Australian Competition and Consumer Commission (ACCC) inquiry into supermarket pricing [42], which may have heightened caution among potential participants regarding disclosure of commercially sensitive information. Additionally, the Australian supermarket sector is highly concentrated, with two major chains dominating market share, which structurally limits the pool of eligible decision-makers, regardless of willingness to participate. Building long-term partnerships with retailers prior to data collection, demonstrating a clear value proposition for participating organisations, and ensuring robust confidentiality protections may improve engagement in future research. Cost-effectiveness was not assessed, despite being identified as a key factor in retailer implementation decisions in previous research. Consumer perspectives are reported for the sample as a whole; variation by age, sex, socioeconomic position and metropolitan or rural residence was not examined, and it is possible that receptiveness to particular strategies differs across population subgroups. Finally, consumer perspectives were operationalised as the perceived likelihood that an initiative would influence purchasing, not as actual purchasing behaviour. Perceived influence and actual behaviour change may differ, and consumer ratings should not be read as estimates of effect, as they may not fully reflect real-world responses.

### 4.2. Conclusions

Pricing and proximity strategies appear to offer the strongest ‘win-win-win’ opportunities identified in this study, showing convergence across scientific evidence, retailer perspectives, and consumer perspectives, though retailer findings should be interpreted as exploratory given the small sample size. The evidence/retailer/consumer alignment model offers a replicable framework for identifying strategies where retailer feasibility, consumer support, and scientific evidence converge. Priority areas for future work include engaging larger and more diverse samples of retailers, and examining whether consumer receptiveness to particular strategies varies by age, socioeconomic position, sex or location to inform targeting. Further work should also explore what drives the gap between evidence and retailer practice for promotion plus, and whether the stakeholder alignment identified here translates into implementation and, ultimately, population-level behaviour change.

## Figures and Tables

**Figure 1:**
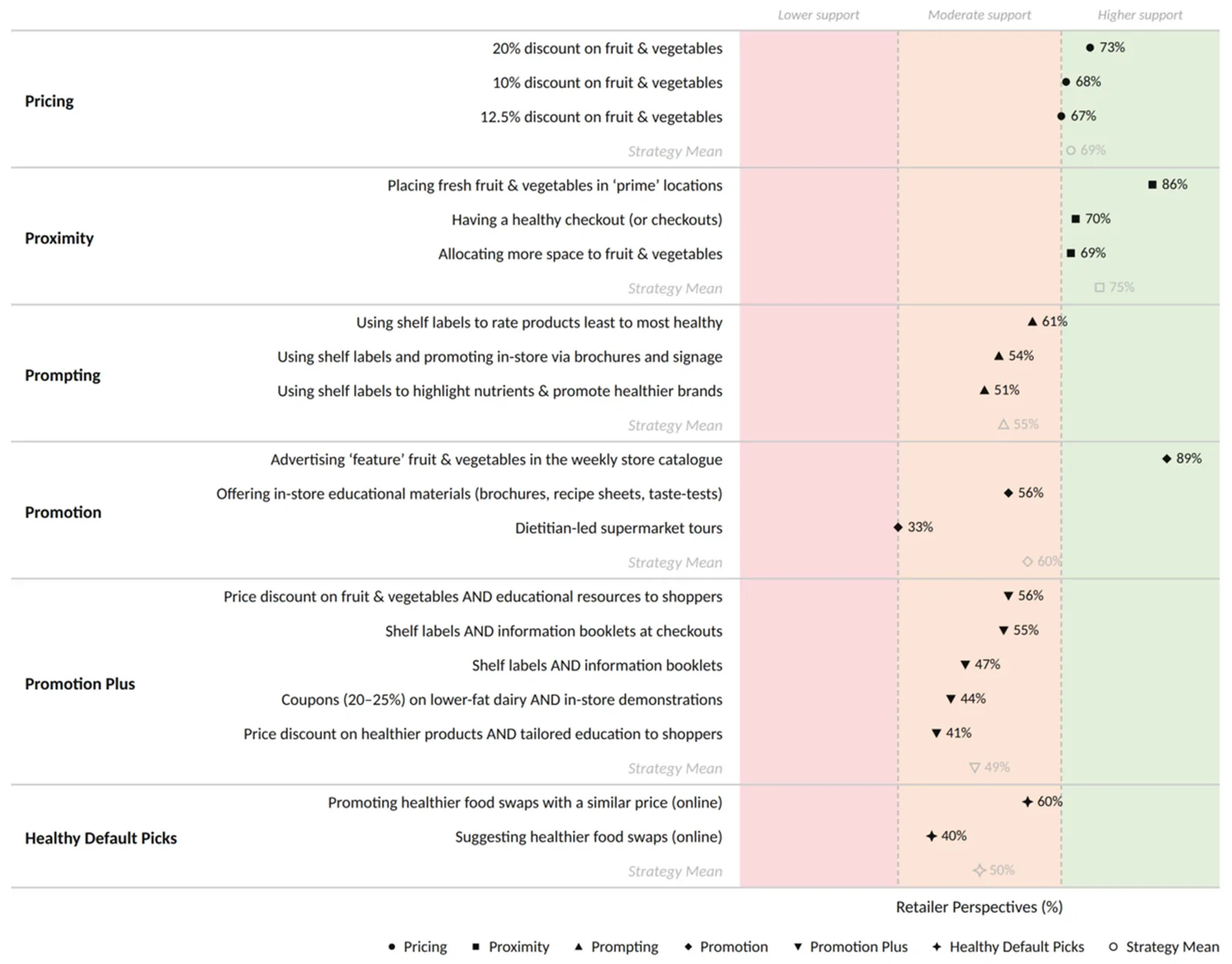
Retailer perspectives (n = 5) on evidence-based healthy supermarket initiatives (n = 19). Note: Each point represents one initiative, plotted against the composite retailer perspectives score (mean of five factors: ease of implementation, effectiveness, scalability, longer-term viability, and willingness to try), expressed as a percentage of the maximum possible score (/6). Hollow markers indicate the mean across the strategy. Strategy means were calculated from unrounded values. Dashed lines indicate thresholds at 33% and 67%, defining lower (red shading), moderate (orange shading), and higher (green shading), respectively. Figure generated by AI, checked and verified for accuracy by the authors.

**Figure 2:**
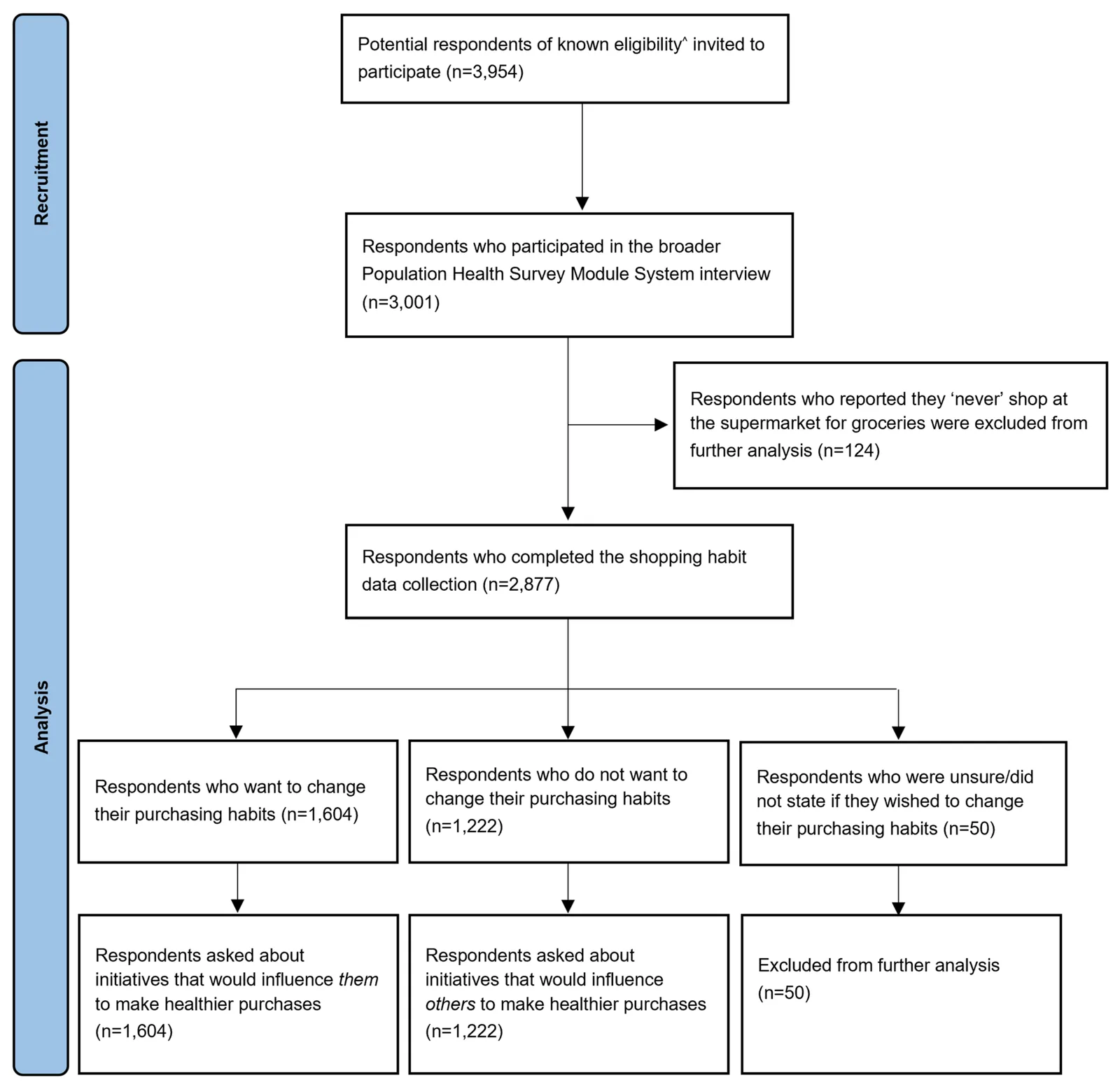
Participant flow diagram. Note: ^^^, known eligibility refers to individuals aged 18 years and over who are residents of South Australia. Numbers are weighted and may not sum exactly to totals due to rounding.

**Figure 3:**
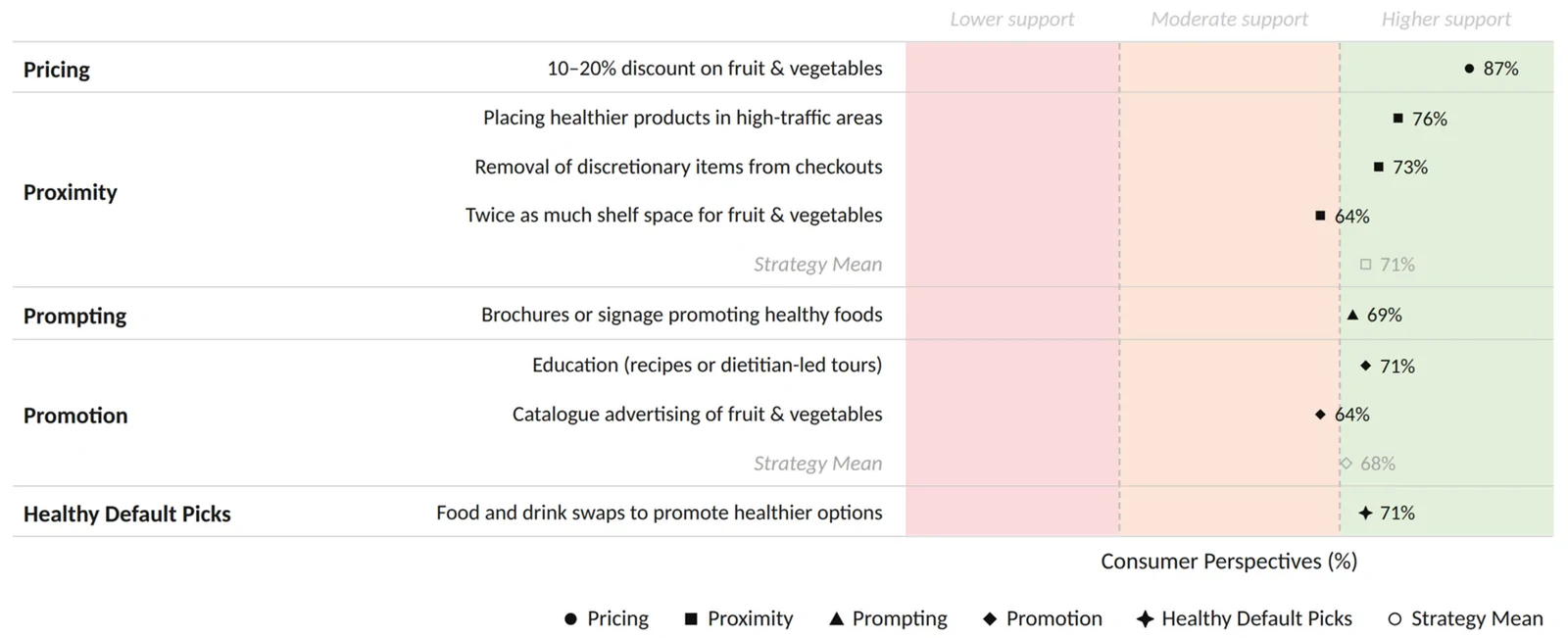
Consumer perspectives (n = 2826) on evidence-based healthy supermarket initiatives. Note: Each point represents one initiative, plotted against the mean consumer perspective score (perceived likelihood of influencing purchasing behaviour), expressed as a percentage of the maximum possible score (/5). Where more than one initiative was presented within a strategy, hollow markers indicate the mean across the strategy. Strategy means were calculated from unrounded values. Dashed lines indicate thresholds at 33% and 67%, defining lower (red shading), moderate (orange shading), and higher (green shading) bands. Figure generated by AI, checked and verified for accuracy by the authors.

**Figure 4:**
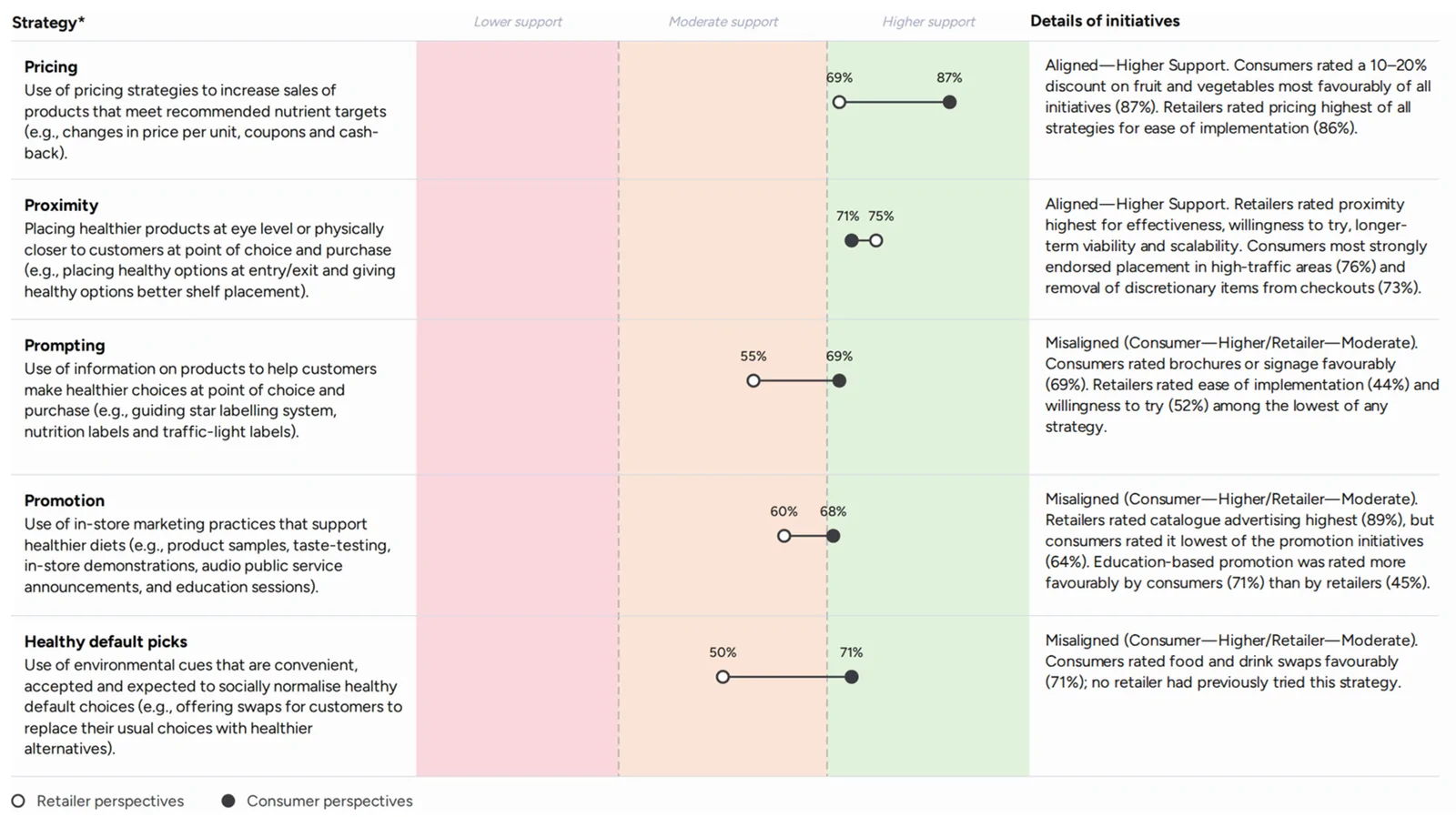
Retailer (n = 5) and consumer perspectives (n = 2826) on evidence-based healthy supermarket strategies. Note: Each strategy is represented by two markers: open markers indicate retailer perspectives, filled markers indicate consumer perspectives. Retailer values are a composite score across five factors (ease of implementation, effectiveness, scalability, longer-term viability, and willingness to try), expressed as a percentage of the maximum possible score (/6). Consumer values are the mean perceived likelihood that a strategy would influence purchasing behaviour, expressed as a percentage of the maximum possible score (/5). Where a strategy comprised more than one initiative, values are the mean across those initiatives (number of initiatives, retailer/consumer: pricing 3/1; proximity 3/3; prompting 3/1; promotion 3/2; healthy default picks 2/1). The two scores are derived from different constructs and are not directly equivalent; the figure summarises the relative positioning of each strategy within each stakeholder group rather than a like-for-like comparison of scores. Dashed lines indicate a priori thresholds at 33% and 67% (thirds of a 0–100% scale), defining lower (red shading), moderate (orange shading), and higher (green shading) bands. Promotion plus is not shown, as it was assessed by retailers only. Given the small retailer sample, retailer values should be interpreted as indicative estimates and the figure as exploratory. * Strategy descriptions adapted from Slapø et al. [22]. Figure generated by AI, checked and verified for accuracy by the authors.

## Data Availability

The data are available upon reasonable request and with the permission of the Commonwealth Scientific and Industrial Research Organisation (CSIRO) and Preventive Health SA.

## Supplementary Materials

The following supporting information can be downloaded at https://www.mdpi.com/article/10.3390/foods15193412/s1. Survey questions included in the Population Health Survey Module System (PHSMS) related to consumers shopping habits and perspectives on supermarket initiatives; Table S1: Description of how initiatives were adapted to present to retailers versus consumers; Table S2: Retailer perspectives (n = 5) on and prior experience with each of the 19 evidence-based initiatives; Table S3: Sociodemographic characteristics of participants from the consumer survey (/2877); Table S4: Shopping habits of participants from the consumer survey; Table S5: Consumer perspectives regarding the influence of initiatives in changing purchasing behaviour by self- and other-referent framing; Figure S1: Retailer perspectives (n = 5) on the 19 evidence-based initiatives; Figure S2: Grocery categories consumers (n = 1604) reported they would like to (A) purchase more of, and (B) purchase less of.
