# Supplementary material for: Healthier Supermarkets: Aligning Retailer and Consumer Perspectives on Evidence-Based Strategies to Improve Purchase Behaviour
Source: Foods. 2026 Sep 24;15(19):3412. doi: 10.3390/foods15193412 (PMC13664380; doi:10.3390/foods15193412)
Supplement: Supplementary file 1 [file foods-15-03412-s001.zip › foods-4544037-supplementary.pdf]

Supplementary Materials

Brooker PG[1], Brindal E[1], Watson-Tran A[2], Hendrie GA[1]. Healthier supermarkets: Aligning retailer and consumer perspectives on evidence-based strategies to improve purchase behaviour

[1]Health and Biosecurity, Commonwealth Scientific and Industrial Research Organisation (CSIRO), PO BOX 10041 Adelaide BC 5000, South Australia, Australia

[2] Preventive Health SA, Adelaide, SA 5000, Australia

Corresponding author:

Paige G Brooker Paige.Brooker@csiro.au

Survey questions included in the Population Health Survey Module System (PHSMS) related to consumers shopping habits and perspectives on supermarket initiatives

Part I: Shopping habits

These next questions are about how you shop for food and beverages in supermarkets. This does not include other retailers such as bakeries, farmers markets, butchers or fruit and vegetable grocers, for example. It also does not include purchasing household or personal care items

When answering these questions, think about the food and beverage shopping you do for yourself or your family

1) **In the last 4-weeks, how often did you shop for food and beverages at the supermarket (either in-store or online)?**
    1. **Daily**
    2. **5-6 times per week**
    3. **3-4 times per week**
    4. **1-2 times per week**
    5. **Once a fortnight**
    6. **Less than once a fortnight**
    7. **Never – I don't shop for food and beverages at the supermarket**
    8. Don't know
    9. Prefer not to say

If Q1 = 7 (never) end survey

2) **When shopping for food and beverages at a supermarket, do you *usually* shop in-store, or online?**
    1. **In-store**
    2. **Online**
    3. **A combination of both**
    4. Don't know
    5. Prefer not to say

3) **Who do you *usually* do the shopping with?**
    1. **Alone**
    2. **Partner**
    3. **Parent or guardian**
    4. **Dependent(s) – children**
    5. **Housemate(s)**
    6. Other ____________________
    7. Don't know
    8. Prefer not to say

4) **Do you shop at any of these supermarkets on a regular basis (i.e., usually, or most of the time)?** *[select all that apply]*
    1. **Aldi**

2. **Coles**
3. **Drakes**
4. **Foodland**
5. **IGA**
6. **Woolworths**
7. **Other ___________________**
8. **No, I shop wherever / I don't have a regular supermarket**
9. Don't know
10. Prefer not to say

If Q4 = 1-7, ask Q5. If Q4 = 8, ask Q6. All respondents then move to Part II, Q7.

5) **Why do you choose (pipe response/these) supermarket/s over other supermarkets?**
   [Open response - Multiple response allowed]
   1. The location is convenient
   2. Accessibility (e.g., parking)
   3. It is the only supermarket nearby
   4. Competitive prices and discounts
   5. Product quality/range/variety
   6. Customer service
   7. Store promotions/loyalty/rewards programs
   8. Emphasis on supporting local
   9. Online shopping and options such as click and collect
   10. Other _ please specify
   11. Don't know
   12. Prefer not to say

6) **Why don't you shop at a regular supermarket?**
   (Open response, multiple response allowed)
   1. I shop wherever is convenient at the time
   2. I shop where it has good accessibility (e.g., parking)
   3. I shop around for competitive prices and discounts
   4. I feel there is a lack of product quality/range/variety from a single store
   5. I shop around for promotions at different stores
   6. I like having different loyalty/rewards programs
   7. No one store has an emphasis on supporting local
   8. Don't know
   9. Prefer not to say

Part II: Health Status, Importance of healthy eating and responsibility

The next questions are about making healthy/healthier food choices.

7) **Thinking about the supermarket environment – i.e., the store layout, the products that are available for sale and how they are promoted - how much do you think the supermarket influences what foods and beverages that you buy?**
    1. **To a great extent**
    2. **Somewhat**
    3. **Neutral**
    4. **Very little**
    5. **Not at all**
    6. Don't know
    7. Prefer not to say

We are interested in whether you want to change the types of foods or beverages you buy in supermarkets.

8) **Generally speaking, do you want to improve the healthiness of foods and beverages you buy?**
    1. **Yes**
    2. **No**
    3. Don't know
    4. Prefer not to say

If Q8 = 2, ask Q9 then move to instructions prior to Q12. If Q8 = 1, ask Q10 and Q11, then move to instructions prior to Q12.

9) **Why not?**
    (Open response, multiple responses allowed)
    1. I think I already eat well enough
    2. I don't care about the healthiness of the food I buy
    3. Healthiness is not a priority right now
    4. I just think about cost (of living) right now – too expensive to buy healthy food and beverages
    5. I just need my family to eat (fussy eaters)
    6. Other ____________________
    7. Don't know
    8. Prefer not to say

10) **Which of the following would you like to purchase *more* of** [*select all that apply*]
    1. **fruit**
    2. **vegetables**
    3. **wholegrain foods – e.g., multigrain/wholemeal breads and cereals**
    4. **healthier (lean) meat, legumes, eggs and/or tofu**
    5. **low-fat dairy (milk, yoghurt, cheese)**
    6. Don't know
    7. Prefer not to say

11) **Which of the following would you like to purchase *less* of** [*select all that apply*]
    1. **cakes and sweet biscuits**
    2. **chocolate and confectionery**
    3. **ice-cream**

4. **processed meat products (e.g., sausages, nuggets, frozen fish)**
5. **savoury pies and pastries**
6. **savoury snacks (e.g., crisps, savoury biscuits)**
7. **sugary or artificially sweetened (‘sugar free’) drinks – e.g., soft drinks, cordials, energy and sports drinks**
8. Don’t know
9. Prefer not to say

If Q8 = 1 (yes) ask Q12 a-h
If Q8 = 2 (no) ask Q13 a-h

Part III: Perceptions about instore initiatives to encourage healthy food choices

The next questions are about your opinion on healthy supermarket initiatives. I'm going to read out a list of initiatives, and I want you to tell me how likely it would encourage *you* to buy healthier foods and beverages.

12a. Having twice as much space for fruit and vegetables

1. **Very likely**
2. **Likely**
3. **Neither likely or unlikely**
4. **Unlikely**
5. **Very unlikely**
6. Don't know
7. Prefer not to say

12b. Promoting fruit and vegetables in the catalogue

1. **Very likely**
2. **Likely**
3. **Neither likely or unlikely**
4. **Unlikely**
5. **Very unlikely**
6. Don't know
7. Prefer not to say

12c. Offering a 10%-20% discount on fruit and vegetables

1. **Very likely**
2. **Likely**
3. **Neither likely or unlikely**
4. **Unlikely**
5. **Very unlikely**
6. Don't know
7. Prefer not to say

12d. Placing healthier products in high traffic areas (e.g., end-of-aisle displays, checkouts etc)

1. **Very likely**
2. **Likely**
3. **Neither likely or unlikely**
4. **Unlikely**
5. **Very unlikely**
6. Don't know
7. Prefer not to say

12e. Using brochures or signage to promote healthy foods

1. **Very likely**
2. **Likely**
3. **Neither likely or unlikely**
4. **Unlikely**

5. **Very unlikely**
6. Don't know
7. Prefer not to say

12f. Offering education to shoppers (e.g., recipes or dietitian-led supermarket tours)

1. **Very likely**
2. **Likely**
3. **Neither likely or unlikely**
4. **Unlikely**
5. **Very unlikely**
6. Don't know
7. Prefer not to say

12g. Using food swaps to promote healthier foods

1. **Very likely**
2. **Likely**
3. **Neither likely or unlikely**
4. **Unlikely**
5. **Very unlikely**
6. Don't know
7. Prefer not to say

12h. Removal of discretionary items like chocolates, lollies, chips and soft drinks, from high traffic areas such as end-of aisle displays and checkouts

1. **Very likely**
2. **Likely**
3. **Neither likely or unlikely**
4. **Unlikely**
5. **Very unlikely**
6. Don't know
7. Prefer not to say

The next questions are about your opinion on healthy supermarket initiatives. I'm going to read out a list of initiatives, and I want you to tell me how likely you think it would encourage *others* to buy healthier foods and beverages.

13a. Having twice as much space for fruit and vegetables

1. **Very likely**
2. **Likely**
3. **Neither likely or unlikely**
4. **Unlikely**
5. **Very unlikely**
6. Don't know
7. Prefer not to say

13b. Promoting fruit and vegetables in the catalogue

1. **Very likely**
2. **Likely**
3. **Neither likely or unlikely**
4. **Unlikely**
5. **Very unlikely**
6. Don't know
7. Prefer not to say

13c. Offering a 10%-20% discount on fruit and vegetables

1. **Very likely**
2. **Likely**
3. **Neither likely or unlikely**
4. **Unlikely**
5. **Very unlikely**
6. Don't know
7. Prefer not to say

13d. Placing healthier products in high traffic areas (e.g., end-of-aisle displays, checkouts etc)

1. **Very likely**
2. **Likely**
3. **Neither likely or unlikely**
4. **Unlikely**
5. **Very unlikely**
6. Don't know
7. Prefer not to say

13e. Using brochures or signage to promote healthy foods

1. **Very likely**
2. **Likely**
3. **Neither likely or unlikely**
4. **Unlikely**
5. **Very unlikely**
6. Don't know
7. Prefer not to say

13f. Offering education to shoppers (e.g., recipes or dietitian-led supermarket tours)

1. **Very likely**

2. **Likely**
3. **Neither likely or unlikely**
4. **Unlikely**
5. **Very unlikely**
6. Don't know
7. Prefer not to say

13g. Using food swaps to promote healthier foods

1. **Very likely**
2. **Likely**
3. **Neither likely or unlikely**
4. **Unlikely**
5. **Very unlikely**
6. Don't know
7. Prefer not to say

13h. Removal of discretionary items like chocolates, lollies, chips and soft drinks, from high traffic areas such as end-of aisle displays and checkouts

1. **Very likely**
2. **Likely**
3. **Neither likely or unlikely**
4. **Unlikely**
5. **Very unlikely**
6. Don't know
7. Prefer not to say

If Q8 = 1 (yes), ask Q14. Otherwise, end survey.

14) If you could only select one healthy supermarket initiative to be implemented, which would it be?

1. **Offering discounts or coupons on selected products**
2. **Offering tours or taste-tests of healthier products**
3. **Reducing the number of discretionary items at checkouts (like chocolates, lollies, chips, and soft drinks)**
4. **Having healthy checkouts (i.e., no discretionary items at checkouts)**
5. **Promoting healthier products in catalogues and signage/brochures**
6. **Having more shelf space for healthier products**
7. **Promoting healthier food swaps**
8. Don't know
9. Prefer not to say

Table S1 Description of how initiatives were adapted to present to retailers versus consumers

Strategy	Initiative – retailer (n=19)	Initiative – consumer (n=8)	Notes
**Proximity**	n=3<br>• Placing fresh fruit and vegetables in 'prime' locations<br>• Allocating more space to fruit and vegetables<br>• Having a healthy checkout (or checkouts)	n=3<br>• Placing healthier products in high traffic areas (e.g., end-of-aisle displays and checkouts etc)<br>• Having twice as much space for fruit and vegetables<br>• Removal of discretionary items from high traffic areas such as end-of-aisle displays and checkouts	Both studies presented 3 proximity initiatives. Consumer wording adapted to reflect the shopper experience rather than retailer implementation. Core concepts are equivalent across studies.
**Pricing**	n=3<br>• Moderate (20%) discount on fruit & vegetables<br>• Small-moderate (12.5%) discount on fruit & vegetables<br>• Small (10%) discount on fruit & vegetables	n=1<br>• Offering a 10%-20% discount on fruit and vegetables	3 discrete discount levels (10%, 12.5%, 20%) presented to retailers were consolidated into 1 representative range (10-20%) for consumers to reduce survey burden within the PHSMS question limit.
**Prompting**	n=3<br>• Using shelf labels to promote healthy products. Rate products from least healthy to most healthy<br>• Using shelf labels to promote healthy products AND promoting the initiative in-store via brochures and signage<br>• Using shelf labels to promote healthy products. Use different variants of shelf labels to highlight nutrients and promote more healthy brands	n=1<br>• Using brochures or signage to promote healthy foods	3 retailer initiatives were condensed to 1 consumer initiative. All three retailer initiatives involve shelf labels, however, 'shelf labels' is retailer terminology that may not be meaningful to consumers in a survey context. The consumer initiative was therefore reworded to 'brochures or signage' to reflect how consumers recognise and experience in-store prompting at the point of purchase. The underlying concept (use of in-store visual information to guide healthier choices) is equivalent across both studies.
**Promotion**	n=3<br>• Supermarket tours	n=2	2 retailer initiatives (supermarket tours; in-store educational materials) were consolidated into 1 broader consumer 'education' initiative. The

	• Offering instore educational materials to shoppers (e.g., brochures, recipe sheets, taste-tests)<br>• Advertising 'feature' fruit and vegetables in the weekly store catalogue	• Offering education to shoppers (e.g., recipes or dietitian-led supermarket tours)<br>• Promoting fruit and vegetables in the catalogue	catalogue initiative is directly comparable across both studies.
**Promotion Plus**	n=5<br>• Placing a small-moderate discount on healthier products AND providing tailored education to shoppers<br>• Placing a moderate discount on fruit and vegetables AND providing educational resources to shoppers<br>• Offering coupons (20-25%) on lower fat dairy products AND providing instore demonstrations<br>• Using shelf labels AND providing information booklets at checkouts<br>• Using shelf labels AND providing information booklets. Use shelf labels to highlight 'healthier' products AND provide information booklets that list 'Special Diet Alert' brand names and specific nutrient values	nil	Promotion Plus was not presented to consumers as a category because consumers encounter individual contextual cues at the point of purchase rather than coordinated multi-component strategies.
**Healthy Default Picks**	n=2<br>• Promoting healthier food swaps with a similar price (online)<br>• Suggesting healthier food swaps (online)	n=1<br>• Using food and drink swaps to promote healthier options	2 retailer initiatives (promoting swaps at a similar price; suggesting swaps automatically) were consolidated into 1 broader consumer 'swaps' initiative. The online framing present in both retailer initiatives was removed for the consumer version, as the PHSMS survey was not specific to online shopping. Core concept is broadly equivalent.

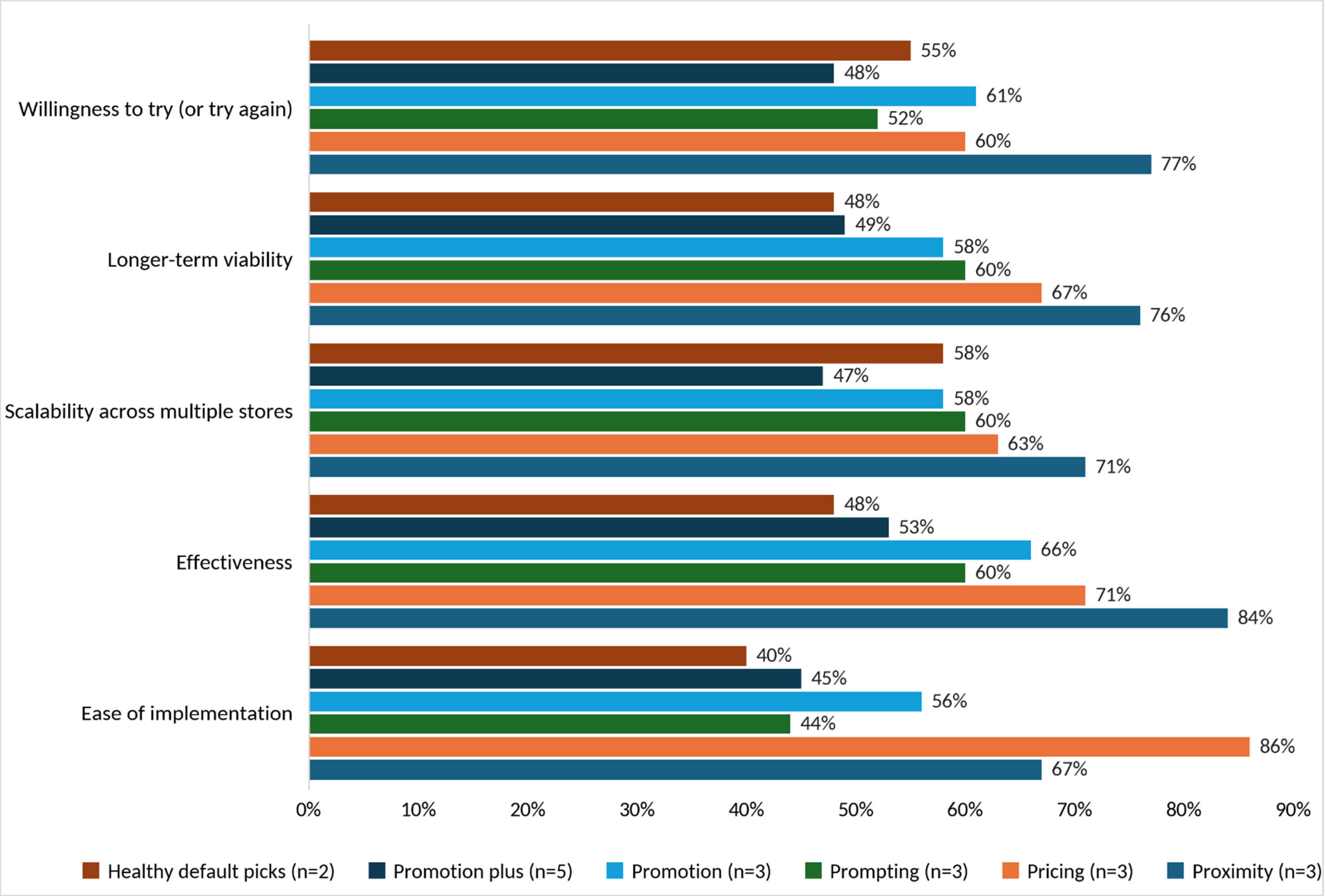

Figure S1 Retailer perspectives (n=5) on the 19 evidence-based initiatives

Note: Bars show the mean rating for each factor across the initiatives within each strategy, rated by five retailer respondents on a six-point scale and expressed as a percentage of the maximum possible score (/6). 'n' in the legend refers to the number of initiatives within each strategy, not the number of respondents.

Table S2 Retailer perspectives (n=5) on and prior experience with each of the 19 evidence-based initiatives

Strategy	Initiative	Composite score (%)	Tried (of 5)
**Proximity**	Placing fresh fruit and vegetables in 'prime' locations	86	5
	Allocating more space to fruit and vegetables	69	3
	Having a healthy checkout (or checkouts)	70	3
	Strategy Mean	75	11 of 15
**Pricing**	Moderate (20%) discount on fruit & vegetables	73	4
	Small-moderate (12.5%) discount on fruit & vegetables	67	3
	Small (10%) discount on fruit & vegetables	68	4
	Strategy Mean	69	11 of 15
**Prompting**	Using shelf labels to promote healthy products. Rate products from least healthy to most healthy	61	2
	Using shelf labels to promote healthy products AND promoting the initiative in-store via brochures and signage	54	2
	Using shelf labels to promote healthy products. Use different variants of shelf labels to highlight nutrients and promote more healthy brands	51	2
	Strategy Mean	55	6 of 15
**Promotion**	Supermarket tours	33	1
	Offering instore educational materials to shoppers (e.g., brochures, recipe sheets, taste-tests)	56	4
	Advertising 'feature' fruit and vegetables in the weekly store catalogue	89	5
	Strategy Mean	60	10 of 15
**Promotion Plus**	Placing a small-moderate discount on healthier products AND providing tailored education to shoppers	41	0
	Placing a moderate discount on fruit and vegetables AND providing educational resources to shoppers	56	2
	Offering coupons (20-25%) on lower fat dairy products AND providing instore demonstrations	44	0
	Using shelf labels AND providing information booklets at checkouts	55	1
	Use shelf labels to highlight 'healthier' products AND provide information booklets that list 'Special Diet Alert' brand names and specific nutrient values	47	1
	Strategy Mean	49	4 of 25
**Healthy Default Picks**	Promoting healthier food swaps with a similar price (online)	60	0
	Suggesting healthier food swaps (online)	40	0
	Strategy Mean	50	0 of 10

Note: composite score is the mean across five factors, expressed as a percentage of the maximum possible score; "Tried" is the number of respondents (of five) whose supermarket had tried that initiative. Strategy means were calculated from unrounded initiative scores and may differ slightly from the mean of the rounded values shown.

Table S3 Sociodemographic characteristics of participants from the consumer survey (/2877)

Characteristics	n (%)
**Sex**	
Female	1,569 (54)
Male	1,307 (45)
**Age, years (Mean ± SD)**	53 ± 17
**Location**	
Metropolitan	2,108 (73)
Rural	769 (27)
**SEIFA**	
Lowest quintile	735 (26)
Low quintile	521 (18)
Middle quintile	581 (20)
High quintile	528 (18)
Highest quintile	511 (18)
**Educational attainment**	
Secondary school or less	1,127 (39)
TAFE, certificate, diploma	1,035 (36)
Degree or higher	699 (24)
Did not report	17 (1)
**Employment status**	
Employed*	1,612 (56)
Unemployed	85 (3)
Other#	1,144 (40)
Did not report	35 (1)
**Marital status**	
Married/Living with partner	1,453 (51)
Separated/Divorced	518 (18)
Widowed	252 (9)
Never Married	602 (21)
Not stated	52 (2)
**Dependents in household**	
0	2,059 (72)
1	336 (12)
2	303 (11)
3+	150 (5)
Not stated	25 (1)

Note: *, Employed included full-time employed, part-time employed and casual employment; #, Other category includes respondents engaged in home duties, unable to work, carers, volunteers, students, retired and any other response. Data with cell counts <5 not shown. Numbers are weighted and may not sum exactly to totals due to rounding. Percentages may not add to 100 due to rounding.

Abbreviations: SD, standard deviation; SEIFA, Socio-Economic Indexes for Areas

Table S4 Shopping habits of participants from the consumer survey^

	n (%)
**Frequency of visits to the supermarket (/3,001)**	
5+ times/week	156 (5)
3-4 times/week	580 (19)
1-2 times/week	1,766 (59)
<1 time/week	372 (12)
Never#	124 (4)
**Shopping mode (/2,877)**	
In-store	2,461 (86)
Online	168 (6)
A combination of both	246 (9)
**Shop with … (/2,877)**	
Alone	1,983 (69)
Partner	538 (19)
Parent or guardian	50 (2)
Dependent(s) - children	158 (5)
Housemate(s)	13 (0)
Other	124 (4)
Don't know	9 (0)
**Regular retailer (/2,877)** *	
No	22 (1)
Yes …	
ALDI	886 (31)
Coles	1,352 (47)
Drakes	686 (24)
Foodland	855 (30)
IGA	332 (12)
Woolworths	1,551 (54)
Other	30 (1)

Note: ^, these findings relate to how respondents shop for groceries (food and beverages) in supermarkets - this does not include other retailers such as bakeries, farmers markets, butchers etc, nor does it include shopping for household or personal care items; #, respondents who reported they 'never' shopped at a supermarket were excluded from any further analysis. Frequency of visits is presented for all respondents who completed the interview (n=3001) to show the full distribution, including those subsequently excluded; percentages reported in the text for this item are based on the analysed sample (n=2877) and therefore differ from those shown here;*, respondents could choose more than one retailer they shop with on a regular basis (i.e., usually or most of the time). Data with cell counts <5 not shown. Numbers are weighted and may not sum exactly to totals due to rounding. Percentages may not add to 100 due to rounding.

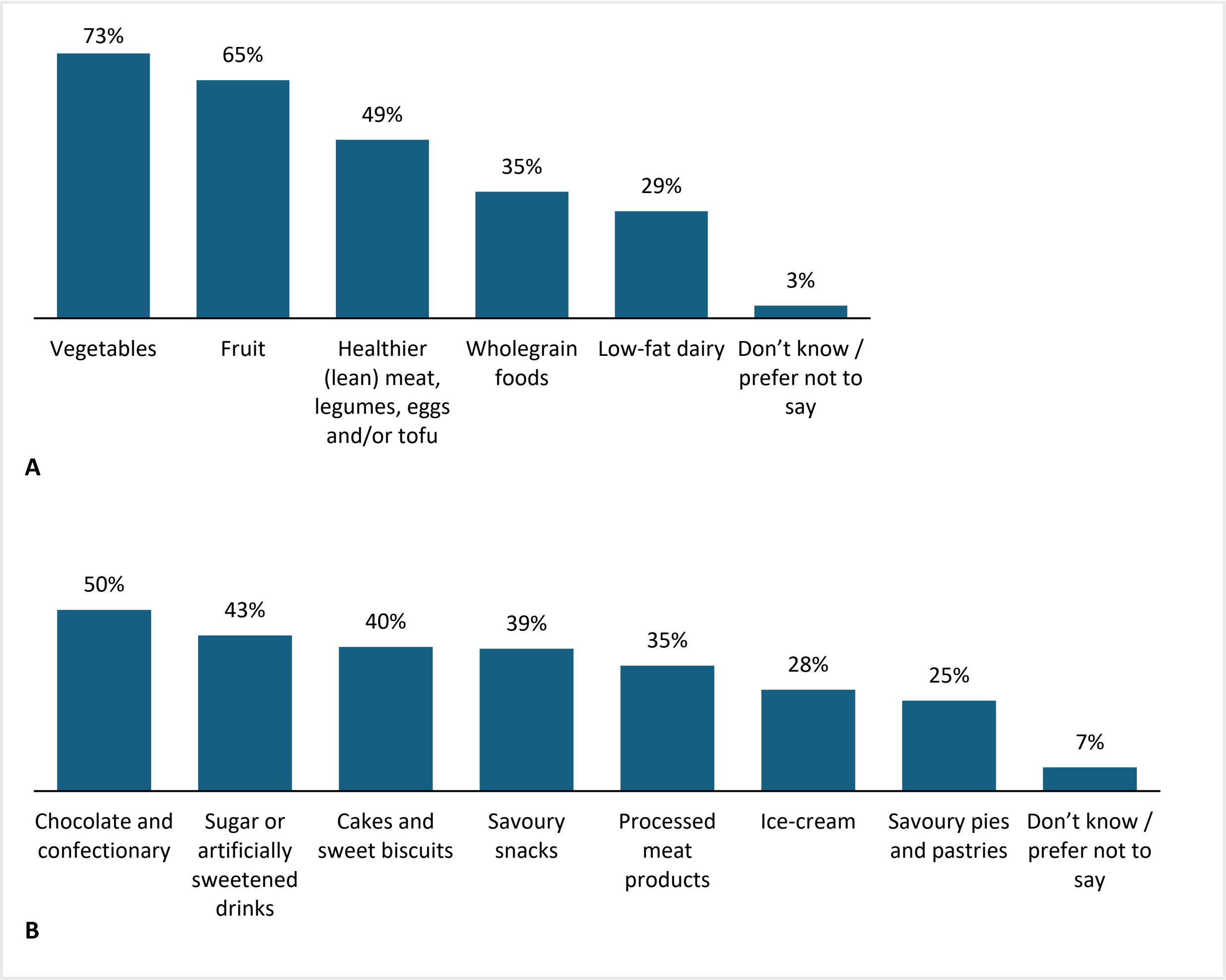

Figure S2 Grocery categories consumers (n=1604) reported they would like to (A) purchase more of, and (B) purchase less of

Table S5 Consumer perspectives regarding the influence of initiatives in changing purchasing behaviour by self- and other-referent framing

	**Self^**	**Others***	**Difference**
**Promotion**			
Promoting fruit and vegetables in the catalogue	63%	66%	3%
Offering education to shoppers (e.g., recipes or dietitian-led supermarket tours)	71%	70%	1%
**Proximity**			
Having twice as much space for fruit and vegetables	67%	61%	6%
Placing healthier products in high traffic areas (e.g., end-of-aisle displays, checkouts etc)	77%	75%	2%
Removal of discretionary items from high traffic areas such as end-of aisle displays and checkouts	74%	73%	1%
**Prompting**			
Using brochures or signage to promote healthy foods	69%	69%	0%
**Pricing**			
Offering a 10%-20% discount on fruit and vegetables	88%	85%	2%
**Healthy Default Picks**			
Using food and drink swaps to promote healthier options	73%	69%	5%

Note: ^, these findings relate to how likely respondents thought the initiative would encourage them to purchase healthier foods and beverages (n=1604); *, these findings relate to how likely respondents thought the initiative would encourage others to purchase healthier foods and beverages (n=1222). Differences were calculated from unrounded values.
